# Free will as structured unpredictability: toward a symbiotic human–AI relationship

**DOI:** 10.3389/frai.2026.1694537

**Published:** 2026-02-13

**Authors:** Mory Ghomshei, Karim C. Abbaspour

**Affiliations:** 1 Carnotech Energy Inc, Vancouver, BC, Canada; 2 2w2e Environmental Consulting GmbH, Dübendorf, Switzerland

**Keywords:** free will in AI, human–AI coexistence, Shannon’s entropy extension, structured unpredictability, symbiotic intelligence

## Abstract

Human history has been shaped by revolutions of varying pace, with artificial intelligence (AI) emerging in mere decades. This paper introduces a preliminary framework for fostering a symbiotic human–AI relationship by integrating human free will into AI systems. We conceptualize free will as ‘structured unpredictability’ and propose a speculative extension of Shannon’s information theory to model its informational contributions. By framing free will as an informational surplus, we envision AI as a mirror and amplifier of human creativity. While theoretical, this framework lays the foundation for future empirical and computational research to preserve human autonomy and diversity in AI-driven systems.

## 1 Introduction

Human beings possess attributes that remain uniquely our own, qualities such as humility, charity, piety, wisdom, and creativity, all rooted in the exercise of free will (Schuon, 1992). While machines share with us certain capabilities-intelligence, responsiveness, learning-these alone cannot capture the distinctively human capacity for autonomous choice. Free will allows us to act beyond deterministic prediction, introducing novelty, meaning, and cultural depth into the world.

Humanity continually navigates the threshold between *order* and *chaos*. Order represents what is known and predictable, chaos, the realm of uncertainty and creative possibility. It is at this frontier that Beethoven’s symphonies, Einstein’s equations, and Rumi’s poetry first emerge, unpredictable sparks later refined into structured knowledge through deliberate human will. For artificial intelligence to truly serve humanity, it must integrate into this dynamic interplay, supporting not just prediction and optimization, but also the human capacity to create, deviate, and project meaning (Human Development Report, 2025; Hao et al., 2025).

The rapid rise of AI intensifies this challenge. Like previous revolutions, from agriculture to industry, AI promises profound transformation. Yet its unprecedented speed risks amplifying modernity’s burdens: excessive regulation, loss of autonomy, and bureaucratic control (Newman et al., 2022; Presuel and Martinez Sierra, 2024).

Deterministic systems may optimize efficiency but can suppress diversity, spontaneity, and dignity-evoking Orwellian futures where machines enforce conformity rather than enabling freedom. The problem is not competition between humans and machines, but misalignment. AI systems excel at optimizing predefined objectives, yet human goals are often ambiguous, evolving, or in tension with one another. Traditional AI methods, grounded in causal determinism, struggle to account for choices driven by aspiration, imagination, or cultural meaning (Reichberg and Syse, 2021). An algorithm may predict the fastest route, but only a human decides to take a scenic detour for reasons of beauty, memory, or ritual.

This tension underscores the importance of free will in human–AI coexistence. Free will introduces ‘structured unpredictability,’ departures from probabilistic expectation that embody cultural, ethical, or creative values. Preserving this space is essential if AI is to enhance rather than diminish human agency.

To address this, we propose a novel, exploratory framework: extending Shannon’s information theory to include a dimension of free will. Just as Shannon’s entropy quantifies uncertainty in communication, a modified entropy could capture the informational surplus generated when human free will deviates from causal prediction. By treating free will as structured unpredictability and framing it as a complementary axis of information, AI can be reimagined not merely as a predictor of likely outcomes but as a mirror that amplifies human creativity and cultural diversity. While this work is theoretical, it offers a conceptual scaffold for integrating human autonomy into AI systems and serves as a starting point for interdisciplinary research, inviting future studies to validate and operationalize this approach in practical AI designs.

## 2 Free will

Free will stands as a cornerstone of human identity and autonomy, underpinning moral responsibility, creativity (Caruso, 2016, 2021), and cultural diversity (Sommers, 2012). The concept is often defined as contingent on the availability of multiple potential courses of action, making alternative possibilities foundational to its very essence. In this incompatibilist view, free will thus cannot coexist with strict determinism, which precludes genuine alternatives. While compatibilists challenge this by redefining freedom in terms of uncoerced action rather than true alternatives, many philosophers—particularly libertarians—argue that free will is essential for practical notions like moral values, creativity, and self-determination (O’Connor and Franklin, 2018).

For the purposes of this paper, we define free will as the capacity for autonomous choice beyond deterministic prediction, expressed not as randomness but as structured unpredictability, decisions that deviate from causal expectation while remaining meaningful in ethical, cultural, or creative terms. This definition is offered as a starting point to facilitate theoretical modeling within AI, with the understanding that future work must refine it through empirical studies of human decision-making and its measurable impacts on AI outputs.

### 2.1 Philosophical perspectives

The tension between free will and determinism has long occupied human thought. Classical philosophers such as Plato (428/427–348/347 BCE/1911) and Aristotle (384–322 BCE/1926) emphasized deliberation and rational choice, grounding freedom in the capacity to act voluntarily rather than by impulse.

Modern thinkers refined this view. Hume (1711–1776/1902) and Hobbes (1588–1679/1656) conceived of freedom as the absence of external constraint, while Kant (1724–1804/2004) elevated it to the will’s capacity to be “a law unto itself,” thereby grounding human dignity in autonomous action.

Contemporary perspectives have extended the free will debate into evolutionary and scientific domains. In game theory, for instance, free will can be framed as the capacity to select among multiple strategies, akin to mixed equilibria that enhance decision-making flexibility. Meanwhile, neuroscientific research posits that neural unpredictability—potentially driven by quantum processes in the brain—may yield adaptive advantages for survival, allowing organisms to evade deterministic predictability (Georgiev, 2021).

Within philosophical discourse, two core contentious claims about free will often emerge. The first is the requirement of “alternative possibilities,” or the genuine ability to do otherwise in identical circumstances. The second concerns free will as the “ultimate source” of our actions, independent of prior causal chains (Caruso, 2016). Central to both is the notion of moral responsibility, an applied concept with profound practical ties to social practices like praise, blame, and obligation (Caruso, 2021).

#### 2.1.1 Structure and unpredictability

The notion of *structured unpredictability* introduces a profound tension at the heart of free will: how can choice be both unconstrained and meaningfully directed? Philosophers have long grappled with this paradox through metaphysical and systems-theoretic lenses.

From an ontological perspective, Spinoza (1632–1677/1985) reconceived freedom not as escape from causation but as self-determination, the capacity of a being to act in accordance with the necessity of its own nature. Here, the “structure” of human will emerges from internal coherence: actions appear unpredictable from an external viewpoint yet remain intelligible from within, molded by personal values, meanings, and aspirations. This aligns with free will as *structured deviation*: an intrinsic order that thwarts external prediction without lapsing into mere randomness.

Complexity theory offers a complementary framework. Haken’s (1977) synergetics and Prigogine’s (1980) theory of dissipative structures demonstrate how order can spontaneously arise from fluctuations and instability in far-from-equilibrium systems. At the “edge of chaos,” such systems achieve a dynamic equilibrium—patterned yet inherently unpredictable. Human cognition and creativity mirror this: our choices are not capricious but emerge from the self-organization of values, memories, and goals. Thus, free will can be seen as consciousness’s ability to channel indeterminacy into purposeful form.

This synthesis positions free will between the poles of deterministic necessity and stochastic randomness. In informational terms, it echoes the interplay of entropy and structure in complex systems: unpredictability fuels novelty, while value-laden orientation ensures coherence. Structured unpredictability thus represents a nonlinear equilibrium between causal order and creative agency—a realm where human action yields an “informational surplus,” transcending mere algorithmic optimization. Unlike linear systems with a single stable state, nonlinear ones afford multiple equilibrium points, or “basins of attraction,” for divergent yet viable choices.

By anchoring this concept in philosophy and complexity science, we transcend the binary of determinism and chance, forging a conceptual bridge to information theory and beyond.

It is important to distinguish *structured unpredictability arising from autonomous agency* from *structure that is externally imposed through modeling or training*. Contemporary AI systems can generate context-sensitive narratives and value-consistent responses by recombining patterns learned from data. Such behavior may appear to reflect preference, memory, or intention, yet the structure guiding these outputs remains entirely exogenous: it is supplied by training corpora, prompt constraints, and optimization objectives rather than by lived experience or self-originating values.

By contrast, human structured unpredictability is endogenous. The coherence of human choice emerges from internally grounded commitments, biographical memory, ethical responsibility, cultural belonging, and aspirational meaning, that are not reducible to statistical inference over past observations. Human action thus introduces informational deviations that are not merely improbable within a model, but *irreducible to the model itself*. This distinction is central to our account of free will: while artificial systems may simulate value alignment at the level of outputs, only human agency supplies the intrinsic normative structure that transforms unpredictability into meaningful choice.

### 2.2 Determinism in AI

In contrast with human vision, artificial intelligence operates within deterministic frameworks. Algorithms map inputs to outputs according to fixed rules, even when stochastic elements are included. A neural network (Christiano et al., 2017), or Fuzzy Logic’s (Ghomshei et al., 2008a,b) prediction, for instance, are always a function of their architecture and training data. This mirrors the principle of causality in physics, where every effect stems from a prior cause.

Probabilistic models relax strict determinism by adding stochastic elements, but they remain fundamentally tied to historical data. As a result, AI excels at prediction and optimization yet struggles to account for choices guided by aspiration, meaning, human foresight or cultural value. An algorithm may predict the fastest route, but it cannot anticipate a driver’s detour to honor a family ritual or to enjoy a festival street, choices that embody free will.

This contrast can be expressed symbolically. Deterministic information may be represented as C (causal), while free will contributes a complementary dimension F , such that the joint informational state resembles a complex term (C+iF) . This anticipates our later proposal to extend Shannon’s entropy, where causal and free will contributions can be integrated into a single formalism.

### 2.3 Operational definition

For this work, we adopt a pragmatic definition of free will tailored to human–AI interaction: Free will is the introduction of structured unpredictability into decision-making-choices that can deviate from causal probability distributions in ways that reflect cultural, ethical, or creative meaning.

This framing allows us to move beyond philosophical abstraction and explore how free will may be quantified as an informational surplus. In the next section, we build on Shannon’s entropy to formalize this integration, opening a pathway for AI systems that not only predict but also adapt to the uniquely human capacity for autonomous, value-driven choice.

## 3 AI and the second law of thermodynamics

The development of AI has far-reaching implications for societies, not only in technological performance but also in its effects on cultural values and human autonomy. One useful lens for examining these effects is the second law of thermodynamics, which describes the natural tendency of systems toward disorder. By analogy, we can view AI systems as tending toward informational homogeneity when left unchecked, unless enriched by ongoing human input.

Just as physical systems evolve toward entropy, AI systems trained iteratively on their own outputs risk converging toward repetitive, uniform results. Without external novelty, particularly the kind introduced by human free will, AI may drift toward predictability and stasis, mirroring thermodynamic equilibrium. This perspective underscores the necessity of integrating human creativity as a counterbalance, keeping AI systems open, diverse, and adaptable.

### 3.1 Shannon’s information and Boltzmann’s entropy theory

Shannon’s (1948) information theory formalized entropy as a measure of uncertainty in communication systems Equation 1:


(1) $$H(X)=-\sum p(x)*\log_{2}(p(x))$$

Where X is a random variable, and p(x) represents the probability of a specific microstate x occurring during the system’s fluctuations. Here, H(X) quantifies the average uncertainty of a system, with higher entropy corresponding to less predictable outcomes. Around the same time, Boltzmann’s statistical mechanics expressed entropy in physics as Equation 2:


(2) $$S=-k*\sum p(E_{j})*\log_{2}(p(E_{j}))$$

Where S is the system’s entropy, k is the Boltzmann constant, $p(E_{j})$ is the probability distribution of the microstate $E_{j}$ , and the sum is taken over all possible states of the system. While Shannon’s entropy measures uncertainty in information, Boltzmann’s measures disorder in physical systems. Their shared mathematical form suggests a deep analogy: both capture the balance between order and unpredictability.

Applied to AI, this analogy reveals both promise and limitation. Deterministic algorithms tend toward homogeneity, compressing variability into narrow predictive patterns. For example:

- A generative model (e.g., DALL-E or Grok) trained repeatedly on its own outputs risks producing increasingly uniform images.
- Recommendation systems can create “filter bubbles” by narrowing content toward prior user behavior.

In such cases, informational entropy declines as predictability overtakes diversity. Yet cultural perspectives complicate this picture. In some societies, uniformity may be valued as harmony (e.g., collectivist traditions), while in others, diversity is prized as freedom (e.g., individualist traditions).

This cultural relativity highlights the importance of human input. AI, left alone, tends toward entropic uniformity; when guided by human values, it can incorporate unpredictability and meaning. In this sense, free will serves as a source of informational renewal, preventing AI from collapsing into equilibrium.

This insight prepares the ground for Section 3.2, where we propose extending Shannon’s entropy itself to formally integrate free will as a complementary dimension of information.

### 3.2 Integrating free will into Shannon’s equation

The preceding sections established that while AI operates within a deterministic framework, human choice often transcends prediction, emerging from imagination, cultural meaning, and creative deviation. To explore this formally, we propose extending Shannon’s entropy equation with a free will component, not as a definitive law but as a conceptual scaffold for modeling human, AI complementarity.

Shannon’s classical entropy captures causal uncertainty Equation 3:


(3) $$H=-\sum p(E_{j})*\log_{2}p(E_{j})$$

where $E_{j}$ represents possible outcomes determined by prior data or causal processes. This captures the “orderly” side of information. Yet, as argued earlier, human decision-making also includes moments of spontaneity, choosing not only from what is most probable or rational, but from what resonates with cultural values, personal aspirations, or sheer imagination.

To reflect this, we introduce a parallel term $F_{k}$ , representing free will contributions, choices not reducible to causal prediction but meaningful within human contexts (e.g., selecting a scenic detour, an Indigenous storytelling motif, or a design inspired by ubuntu). The extended entropy becomes Equation 4:


(4) $$H=-\sum [p(E_{j})*\log_{2}p(E_{j})+i\;p(F_{k})*\log_{2}p(F_{k})]$$

**Figure 1:**
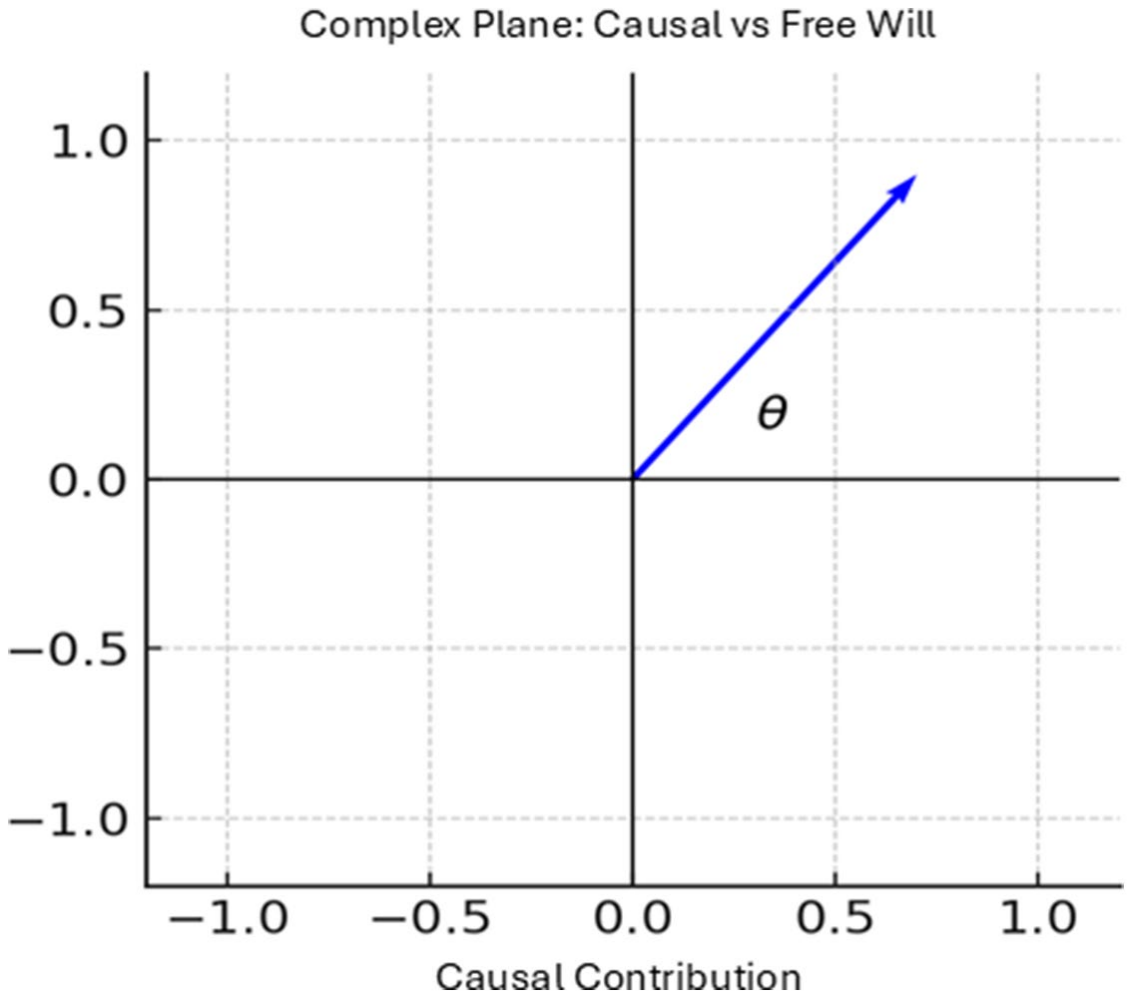
Complex plane showing causal (real axis) and free will (imaginary axis) contributions. The angle *θ* represents their relative balance.

Here the imaginary uniti symbolically distinguishes causal contributions (real axis) from free will contributions (imaginary axis). This does not claim free will is literally imaginary; rather, it acknowledges that human spontaneity occupies a qualitatively different dimension of informational space, one that AI alone cannot exhaust (Figure 1). This extension is a conceptual proposal, intended to illustrate the potential of modeling free will as an informational dimension. While not yet computationally implemented, it provides a theoretical framework for future research to develop practical algorithms and validate its utility in AI systems.

To visualize this interplay, we adopt an exponential form Equations 5–7:


(5) $$H=-r*e^{\mathrm{i\theta}}$$

with


(6) $$r=\sqrt{{\left(\sum p(E_{j})*\log_{2}p(E_{j})\right)}^{2}+{\left(\sum p(F_{k})*\log_{2}p(F_{k})\right)}^{2}}$$

(7) $$\theta =\tan^{-1}(\frac{\sum p(F_{k})*\log_{2}p(F_{k})}{\sum p(E_{j})*\log_{2}p(E_{j})})$$

**Figure 2:**
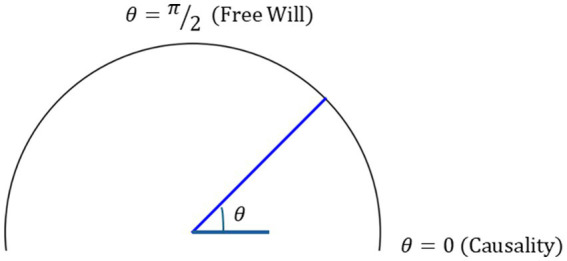
Balance dial illustrating *θ* as the degree to which free will or causality dominates.

Here, r represents the combined “magnitude” of causal and free will contributions, while θ represents their relative balance. When θ→0 , causality dominates (e.g., a recommendation system predicting movies by past viewing history). When θ→π/2 , free will dominates (e.g., a user creating art in DALL-E or Grok inspired by ancestral narratives rather than training data patterns) (Figure 2).

To capture divergence between machine predictions and human choice, we can adopt the Kullback–Leibler divergence (Kullback and Leibler, 1951) Equation 8:


(8) $$D_{\mathrm{KL}}(P\|Q)=\sum P(x_{i})\;\log_{2}(\frac{P(x_{i})}{Q(x_{i})})$$

**Figure 3:**
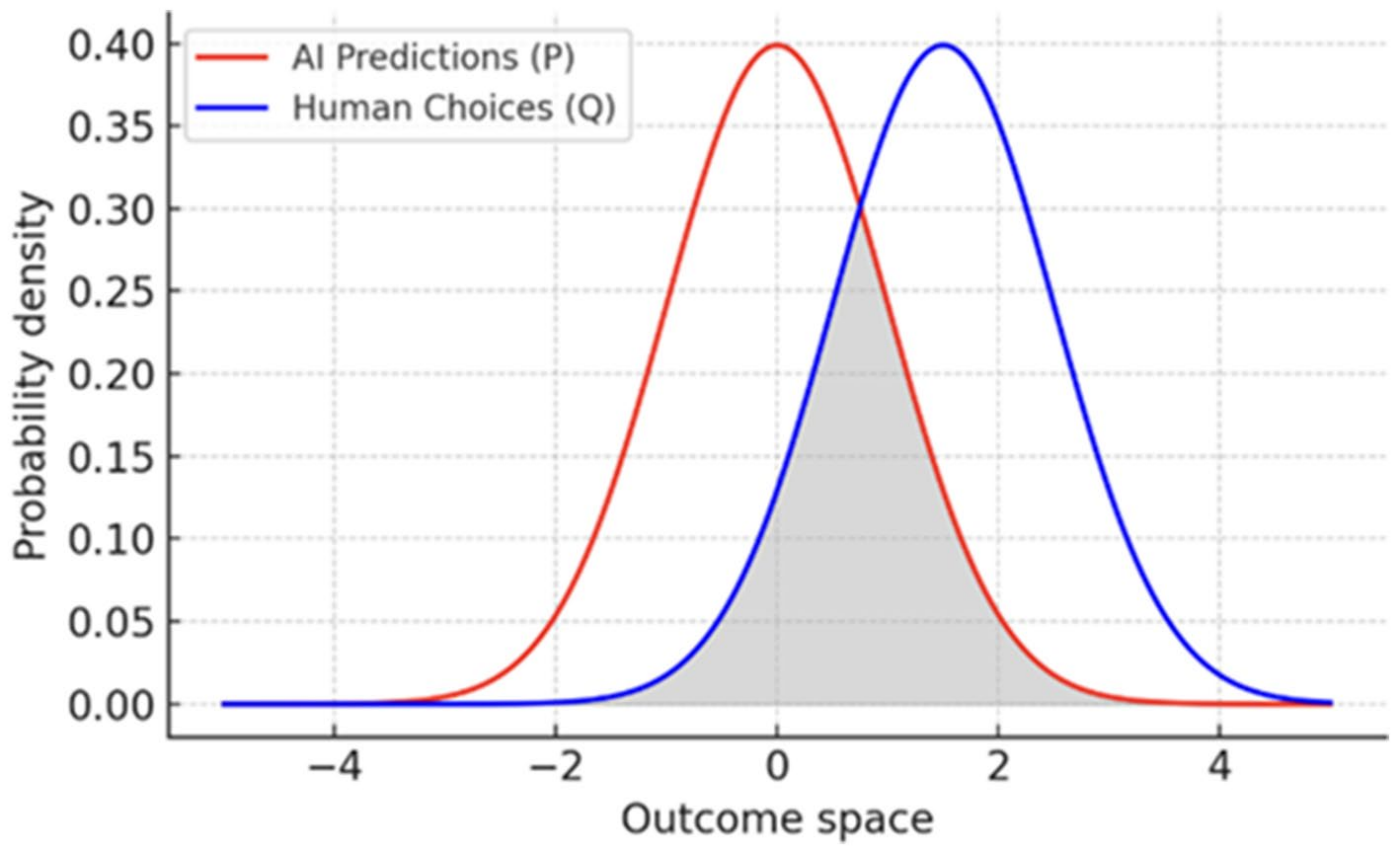
KL divergence illustrating divergence between AI-predicted outcomes (*P*) and human-chosen outcomes (*Q*).

Where $P(x_{i})$ represents a distribution of AI-predicted outcomes and $Q(x_{i})$ the distribution of human choices, A high $D_{\mathrm{KL}}$ indicates that humans introduce genuinely novel information-cultural, aesthetic, or ethical, that AI alone would not generate. These formulations are exploratory, serving as a proof of concept for quantifying the informational surplus created by free will. For instance, an AI system might predict the fastest driving route (P) , while a human detours through a festival street for symbolic or communal reasons (Q) . Likewise, a generative model may default to generic imagery, whereas a human draws on motifs of Japanese harmony, African ubuntu, or Western individualism. In both cases, the divergence between P and Q marks the additional layer of meaning supplied by human agency (Figure 3). Future work will focus on operationalizing these equations, testing them in real-world AI applications, and refining their mathematical rigor.

This framework remains theoretical and exploratory: it does not aim to reduce free will to mere mathematics but to quantify its informational footprint more precisely. By framing free will as an additional dimension of entropy, we highlight how human choices infuse AI outputs with richness—projecting cultural values and creativity onto otherwise neutral algorithmic canvases. Far from mere noise, free will embodies structured unpredictability, fostering openness to novelty, adaptability, and diversity in human–AI collaborations. The proposed extension of Shannon’s entropy thus functions as a conceptual tool, illuminating the dynamic interplay between causal determinism and volitional agency in these systems. Though not yet computationally realized, it lays groundwork for future inquiry: designing algorithms that weave in human creativity, validating the model through empirical tests, and honing its mathematical underpinnings for real-world applicability.

These formulations, while exploratory, bridge philosophical introspection and mathematical modeling with deliberate intent. To elevate structured unpredictability beyond metaphor, however, its informational dynamics demand operationalization—grounded in quantifiable metrics and benchmarked against established paradigms in neuroscience and AI. The ensuing section charts pathways for this formalization, exploring how an extended entropy measure might mature into a robust, empirically testable framework.

### 3.3 Toward a formal framework

While the proposed extension of Shannon’s entropy introduces a symbolic scaffold for integrating free will into information theory, its full value lies in the potential for mathematical specification and empirical validation. This section outlines preliminary directions for formalization and situates the framework within existing theories of information and autonomy.

#### 3.3.1 Operationalization and simulation

The complex entropy formulation can be decomposed into two measurable components:

1. *Causal Entropy (H_C_)*, representing predictive uncertainty derived from historical data, or probabilistic models, and.
2. *Volitional Entropy (H_F_)*, representing systematic deviations from such predictions that reflect intentional, cultural, or creative choice.

An operational measure of 𝐻_𝐹_ may be derived from behavioral or generative datasets in which humans interact with AI systems. Examples include creative design platforms, route-planning tasks, or collaborative text generation. Here, 𝐻_𝐹_ could be estimated as the ‘informational divergence unexplained by predictive models’, normalized by contextual relevance metrics (e.g., semantic or aesthetic alignment scores). This would quantify ‘structured unpredictability’ as a measurable informational surplus arising from human intention.

#### 3.3.2 Relation to existing frameworks

The proposed approach intersects with, but diverges from, several contemporary frameworks addressing autonomy and information:

- *The Free Energy Principle*—Friston (2010) frames cognition as the minimization of surprise through Bayesian inference. Our model complements this by emphasizing positive informational surplus, the deliberate generation of meaning and novelty beyond equilibrium states. Whereas Friston’s principle reduces free energy to maintain stability, structured unpredictability introduces controlled disequilibrium as a source of creativity.
- *Causal Emergence*—Hoel (2017) shows that macro-level causal structures can exhibit greater effective information than their micro-level constituents. Within our framework, free will can be viewed as such a macro-level emergent property: an organizing principle that increases the dimensionality of informational space from 𝐶 (causal) to (𝐶 + 𝑖𝐹), thereby expanding the system’s representational depth.
- *Enactive Cognition*—(Varela et al., 1991) conceives cognition as a process of enactment, the active bringing-forth of a meaningful world through embodied interaction. This resonates with our view that free will is not external to informational flow but an intrinsic modulation within it: a capacity to transform indeterminacy into meaningful structure.

By situating structured unpredictability within this triad of frameworks, we link it to established theories while emphasizing its unique contribution: modeling the generative role of unpredictability in human–AI co-adaptation.

#### 3.3.3 Future mathematical directions

Future work will seek to formalize the complex entropy model using a Hilbert-space representation Equation 9:


(9) $$H=H_{C}+iH_{F}$$

where the magnitude ${\left\|H\right\|}^{2}=H_{C}^{2}+H_{F}^{2}$ defines total informational potential. This representation preserves additivity while distinguishing between causal and volitional contributions. The phase term *θ*, already introduced in Section 3.2, can then be interpreted as a measure of volitional coherence, analogous to phase coherence in quantum information theory (Vedral, 2010). The term *θ* can be considered as a useful complementary informative element, to be preserved in information processing and conveyed to the receivers of information.

Such an approach invites simulation using datasets that combine human and AI outputs, for instance, comparing machine-generated predictions (𝐻_𝐶_) with human-modulated results (𝐻_𝐹_) to quantify the informational surplus attributable to free will. Over time, this may yield a measurable parameter for “autonomy entropy,” linking human creativity and machine optimization within a unified informational framework.

By embedding symbolic extension within a mathematically tractable space and aligning it with existing principles, structured unpredictability can evolve from philosophical metaphor to operational construct, one capable of informing future empirical research on human–AI complementarity.

#### 3.3.4 Toy example and derivation

To illustrate how complex-valued entropy acquires an operational meaning in real decision contexts, consider again the navigation scenario introduced earlier. The driver’s observed trajectory reflects two interacting influences:

1. *Causal structure*: Constraints arising from traffic patterns, geography, or time-of-day regularities.
2. *Volitional structure*: Value-laden, culturally shaped, or idiosyncratic preferences that introduce *structured unpredictability* beyond what a causal model can infer.

Following Sections 2.2–2.4, let *θ* denote a *latent decision state* that combines both influences. We approximate its posterior with a variational density q(θ) .

#### 3.3.5 Complex free-energy formulation

We decompose the variational objective into a ‘causal’ component and a ‘volitional’ component Equation 10:

##### 3.3.5.1 Causal free-energy term


(10) $$F_{C}(q)=E_{q(\theta )}\;[\log q(\theta )-\log p_{\text{causal}}(o,\theta )]$$

This is the usual variational free energy bounding causal surprisal (Equation 11).

##### 3.3.5.2 Volitional divergence term


(11) $$F_{F}(q)=D_{\mathrm{KL}}(q(\theta )∥p_{\text{volitional}}(\theta ))$$

where $p_{\text{volitional}}(\theta )$ is a ‘value-neutral prior’ encoding efficiency-based routes (no volitional structure). We combine these into a ‘complex free energy’ Equation 12:


(12) $${ℱ}_{\mathbb{C}}(q)=F_{C}(q)+i\;F_{F}(q)$$

The ‘real part’ quantifies causal structure and the ‘imaginary part’ quantifies volitional surplus, the portion of behavior not explained by causal constraints.

#### 3.3.6 Toy numerical illustration

Suppose the AI’s causal model accounts for C bits of predictive information (e.g., traffic-based constraints), while the user’s idiosyncratic detour introduces V bits of volitional divergence. Then the complex free energy is Equation 13:


(13) $${ℱ}_{\mathbb{C}}=C+\mathrm{iV}$$

Its ‘magnitude’ is Equation 14:


(14) $$|F_{\mathbb{C}}|=\sqrt{C^{2}+V^{2}}$$

and its ‘phase’ (which we standardize as *φ*) is Equation 15:


(15) $$\phi =\arctan(\frac{V}{C})$$

A representative example yields $\phi \approx 0.59\;\mathrm{rad}\approx 34^{\circ }$ , indicating a noticeable tilt toward volitional influence.

In an interactive navigation system, this phase encodes the ‘balance’ between causal and volitional structure. A shift toward the imaginary axis alerts the AI that the user’s trajectory reflects value-laden or preference-driven decisions rather than pure efficiency.

#### 3.3.7 Derivational note

The imaginary term emerges naturally once the entropy functional is extended into the complex domain: $F_{F}(q)\;$ acts as an orthogonal perturbation to standard variational updates. This prevents collapse into ‘informational homogeneity’ (cf. Section 3.1), enabling richer exploration aligned with human intention.

#### 3.3.8 Refinement via deconvolution: coupling coefficient *α*

Causal and volitional influences may partially overlap. For example, a scenic detour may coincide with festival patterns that also appear in causal traffic data. To account for such interactions, we model the observed latent decision density as a ‘convolution’ Equation 16:


(16) $$p(\theta )=(p_{E}*p_{F})(\theta )$$

Where $p_{E}$ encodes causal latent structure, and $p_{F}\;$ encodes volitional latent structure.

Using Wiener deconvolution on logged interaction trajectories (FFT-based, O(nlogn) ), we recover approximations to $p_{E}$ and $p_{F}$ . We then compute their ‘normalized overlap’:


ρ(E,F)≔correlation or overlap between recoveredcausal and volitional components.

We define the ‘volitional novelty coefficient’ Equation 17:


(17) α≔1−ρ(E,F)

Thus: *α* ≈ 0 implies behavior is almost fully explainable by the causal model, and α ≈ 1 implies behavior expresses strong volitional novelty. We then refine the complex free energy accordingly Equation 18:


(18) $${ℱ}_{\mathbb{C}}^{\left(\alpha \right)}(q)=F_{C}(q)+i\;\alpha \;F_{F}(q)$$

As an example consider that deconvolution reveals ‘40% predictive overlap’ between causal and volitional structure, then: ρ=0.4,α=0.6 . The imaginary term is scaled accordingly, indicating that while the user deviates from the AI’s predicted route, nearly half of this deviation is still grounded in explainable causal structure. This allows finer inference: the system detects ‘irreducible volitional surplus’ without misclassifying environmentally induced deviations as ‘pure free will.’

The implications of this is that large values of the imaginary component ∣Im(ℱℂ)∣ , or α-weighted divergence between human and model predictions, serve as ‘reliable indicators of autonomous human input’. This enables adaptive reinforcement in hybrid navigation systems, creative-collaboration tools, and decision-support interfaces.

## 4 A symbiotic human–AI relationship

Humans and artificial intelligence differ fundamentally in their mode of agency and orientation. Humans act feedforward, guided by experience, aspiration, and imagination. Our capacity to conceive possibilities, desire futures that do not yet exist, and initiate action toward them is rooted in free will understood here as structured unpredictability. Artificial intelligence, by contrast, operates feedback-driven, grounded in historical data, predefined objectives, and optimization procedures. Even when AI systems generate novel or context-sensitive outputs, the goals and values guiding those outputs remain externally supplied rather than internally originated. In the present framework, this distinction, machines interpret and optimize, while humans conceive, desire, and initiate, is adopted as a *normative axiom* rather than a settled empirical claim. It serves to anchor the complementary roles assigned to human agency and artificial systems within a symbiotic human–AI relationship.

Already, AI enhances healthcare, transportation, education, and environmental management. Yet these advances also reshape economies and labor and raise ethical challenges involving privacy, fairness, accountability, and the preservation of human values. Beyond economics and ethics, AI prompts deeper reflection on human uniqueness: if machines replicate aspects of intelligence, what remains distinctively human? We argue it is free will, the capacity to deviate meaningfully from determinism while projecting cultural, ethical, and aspirational meaning. Preserving this capacity, while embedding ethical safeguards into AI design, ensures that AI remains a mirror and amplifier of human values rather than an autonomous force divorced from them. The goal, ultimately, is coexistence. By balancing human free will with responsible AI development, humans and machines can form a partnership in which efficiency and scale are complemented by novelty and meaning, together advancing futures neither could achieve alone.

## 5 Epilogue

Once upon a time, humanity imagined itself in a paradise of abundance, where every need was met without effort. Only one prohibition existed, yet even this boundary proved irresistible. In reaching for the forbidden fruit, humans revealed a deeper truth: we are a species unwilling to live within absolute rules. Constraint breeds revolt, and freedom, however costly, remains our defining essence.

Today, as we stand at the threshold of an AI age, this story echoes anew. The danger is not only that machines may grow powerful enough to shape our destiny, but that humans may descend to the level of machines, trading autonomy for efficiency, becoming bound by the deterministic logic we ourselves have created. Regulation, while necessary for technology, cannot be allowed to become a cage for human aspiration.

The solution lies in reversal: let us discipline machines while unleashing human potential. By embedding ethical safeguards in AI and simultaneously reclaiming our own capacity for free will, we preserve the space for human unpredictability, creativity, and meaning. In this balance, machines remain tools, powerful partners, but never masters.

As Nietzsche observed, “Free will without fate is just as unthinkable as spirit without reality, good without evil. Only antithesis creates the quality.” Against the deterministic fate of machines, human free will discovers its true Icarian flight, rising above the maze of predictability, daring to venture into realms machines can never reach.

## 6 Future work

This paper introduces a theoretical framework for integrating free will into AI systems, setting the stage for deeper exploration. Future research will focus on: (1) empirical validation through computational experiments, such as applying the extended entropy model to generative AI systems; (2) developing methodologies to quantify free will contributions using behavioral data; (3) refining the mathematical framework to ensure computational feasibility; and (4) collaborating with philosophers, AI researchers, and ethicists to address practical and ethical challenges. These efforts will aim to translate our speculative proposal into actionable AI designs that enhance human autonomy and creativity.

## Data Availability

The original contributions presented in the study are included in the article/supplementary material, further inquiries can be directed to the corresponding authors.

## References

[ref1] Aristotle (1926). Nicomachean ethics (Translated by H. Rackham. Loeb classical library 73). Cambridge, MA: Harvard University Press. Available online at: https://www.loebclassics.com/view/LCL073/1926/volume.xml (Accessed November 5, 2025).

[ref2] Caruso G. D. (2016). Free will skepticism and the question of creativity: creativity, desert, and self-creation. Ergo, Open Access J. Philo. 3, 591–607. doi: 10.3998/ergo.12405314.0003.023

[ref3] Caruso G. D. (2021). Skepticism about moral responsibility, the Stanford encyclopedia of philosophy, ed. Zalta E. N.. Stanford University. Available online at: http://plato.stanford.edu/archives/sum2021/entries/skepticism-moral-responsibility/ (Accessed November 5, 2025).

[ref4] Christiano P. F. Leike J. Brown T. B. Martic M. Legg S. Amodei D. (2017). Deep reinforcement learning from human preferences. 31st Conference on Neural Information Processing Systems (NIPS 2017), Long Beach, CA. Available online at: https://proceedings.neurips.cc/paper_files/paper/2017/file/d5e2c0adad503c91f91df240d0cd4e49-Paper.pdf

[ref5] Friston K. (2010). The free-energy principle: a unified brain theory? Nat. Rev. Neurosci. 11, 127–138. doi: 10.1038/nrn2787, 20068583

[ref6] Georgiev D. D. (2021). Quantum propensities in the brain cortex and free will. Biosystems 208:104474. doi: 10.1016/j.biosystems.2021.104474, 34242745

[ref7] Ghomshei M. M. Meech J. A. Naderi R. (2008a). “Fuzzy logic in a postmodern era” in Forging new Frontiers: Fuzzy pioneers II. Studies in fuzziness and soft computing, vol. 218. eds. Nikravesh M. Zadeh A. (Berlin, Heidelberg: Springer).

[ref8] Ghomshei M. Meech J. Naderi R. (2008b). War, peace, and fuzzy logic. Cybern. Syst. 39, 113–135. doi: 10.1080/01969720701853418

[ref9] Haken H. (1977). Some aspects of synergetic. Springer series in synergistics, vol. 2. Berlin, Heidelberg: Springer.

[ref10] Hao X. Demir E. Eyer D. (2025). Beyond human-in-the-loop: sensemaking between artificial intelligence and human intelligence collaboration. Sustain. Futures 10:101152. doi: 10.1016/j.sftr.2025.101152

[ref11] Hobbes T. (1656). The questions concerning liberty, necessity, and chance. Dublin: Andrew Crook.

[ref12] Hoel E. P. (2017). When the map is better than the territory. Entropy 19:188. doi: 10.3390/e19050188

[ref13] Human Development Report. (2025). A matter of choice: people and possibilities in the age of AI. The United Nations Development Programme 1 UN Plaza, New York, NY. Available online at: https://www.undp.org/sites/g/files/zskgke326/files/2025-05/human_development_report_2025.pdf (Accessed November 5, 2025).

[ref14] Hume D. (1902) in Enquiries concerning human understanding and concerning the principles of morals. ed. Selby-Bigge L. A. (Oxford: Clarendon Press), 165.

[ref15] Kant I. (2004). Critique of practical reason (T. K. Abbott, Trans.). Mineola, New York: Dover Publications.

[ref16] Kullback S. Leibler R. A. (1951). On information and sufficiency. Ann. Math. Stat. 22, 79–86. doi: 10.1214/aoms/1177729694

[ref17] Newman J. Mintrom M. O'Neill D. (2022). Digital technologies, artificial intelligence, and bureaucratic transformation. Futures 136:102886. doi: 10.1016/j.futures.2021.102886

[ref18] O’Connor T. Franklin C.E. (2018). Free will. Stanford encyclopedia of philosophy. Available online at: https://philpapers.org/rec/OCOFW

[ref19] Plato (1911). Plato's Phaedo (J. Burnet, Trans). Oxford: Clarendon Press.

[ref20] Presuel R. C. Martinez Sierra J. M. (2024). The adoption of artificial intelligence in bureaucratic decision making: a Weberian perspective. Digit. Gov. Res. Pract. 5:6. doi: 10.1145/3609861

[ref21] Prigogine I. (1980). From being to becoming: time and complexity in the physical sciences. San Francisco: W. H. Freeman & Co.

[ref22] Reichberg G. M. Syse H. (2021). “Applying AI on the battlefield: the ethical debates” in Robotics, AI, and humanity. eds. Braun J. Archer M. Reichberg G. M. Sánchez Sorondo M. (Cham: Springer).

[ref23] Schuon F. (1992). The play of masks. Bloomington: World Wisdom Books Inc.

[ref24] Shannon C. E. (1948). A mathematical theory of communication. Bell Syst. Tech. J. 27, 379–423. doi: 10.1002/j.1538-7305.1948.tb01338.x

[ref25] Sommers T. (2012) Relative justice: cultural diversity, free will, and moral responsibility. Princeton, New Jersey: Princeton University Press. Available online at: http://www.jstor.org/stable/j.ctt7s21b (Accessed November 5, 2025).

[ref26] Spinoza B. (1985). The ethics and selected letters (S. Shirley, Trans.). Indianapolis, Indiana: Hackett Publishing Company.

[ref27] Varela F. J. Thompson E. Rosch E. (1991). The embodied mind: cognitive science and human experience. Cambridge, MA: MIT Press.

[ref28] Vedral V. (2010). Decoding reality: the universe as quantum information. Oxford, UK: Oxford University Press.

